# Butyrylcholinesterase Predicts Cardiac Mortality in Young Patients with Acute Coronary Syndrome

**DOI:** 10.1371/journal.pone.0123948

**Published:** 2015-05-01

**Authors:** Patrick Sulzgruber, Lorenz Koller, Thomas Reiberger, Feras El-Hamid, Stefan Forster, David-Jonas Rothgerber, Georg Goliasch, Johann Wojta, Alexander Niessner

**Affiliations:** 1 Division of Cardiology, Department of Internal Medicine II, Medical University of Vienna, Vienna, Austria; 2 Division of Gastroenterology and Hepatology, Department of Internal Medicine III, Medical University of Vienna, Vienna, Austria; 3 Edwin L. Steele Laboratory, Dept. of Radiation Oncology, Massachusetts General Hospital and Harvard Medical School, Boston, Massachusetts, United States of America; University Heart Center Freiburg, GERMANY

## Abstract

**Background:**

The incidence of acute coronary syndrome (ACS) in young people (≤65 years) is continuously rising. While prognostic factors in ACS are well-investigated less attention has been paid to their age-dependent prognostic value and their particular relevance in younger patients. The aim of our study was to assess the age-dependent prognostic impact of butyrylcholinesterase (BChE).

**Methods:**

Retrospective cohort study including 624 patients with ACS. Patients were stratified by age into equal groups (n = 208) corresponding to “young patients” (45–64 years), "middle-aged patients” (65–84 years) and “old patients” (85–100 years). Cox regression hazard analysis was used to assess the influence of BChE on survival.

**Results:**

After a mean follow-up time of 4.0 (interquartile range [IQR] 2.0–6.4) years, 154 patients (24.7%) died due to a cardiac cause. In the overall cohort, BChE was indirectly associated with cardiac mortality-free survival (adjusted hazard ratio (HR): 0.70 (95% confidence interval [CI] 0.53–0.93, p = 0.01). The primary-analysis of BChE by age strata showed the strongest effect in the age group 45–64 years with an adjusted HR per 1-SD of 0.28 (95% CI 0.12–0.64, p = 0.003), a weaker association with mortality in middle aged (65–84 years: adjusted HR per 1-SD 0.66 [95% CI: 0.41–1.06], p = 0.087), and no association in older patients (85–100 years: adjusted HR per 1-SD 0.89 [95% CI: 0.58–1.38], p = 0.613).

**Conclusion:**

BChE is a strong predictor for cardiac mortality specifically in younger patients with ACS aged between 45 and 64 years. No significant association of BChE with cardiac-mortality was detected in other age classes.

## Introduction

Acute coronary syndrome (ACS) represents the most severe consequence of atherosclerosis, and is associated with substantial morbidity and mortality.[1] Today, the comprehensive knowledge of cardiovascular risk factors enables individual risk stratification in patients with coronary artery disease (CAD). The improvement of long-term risk prediction is crucial for secondary prevention in patients with ACS, in order to reduce hospital re-admission-rates and all-cause mortality.[2] Recently butyrylcholinesterase (BChE) turned out as a strong predictive value.[3–4] It represents a liver-specific enzyme, that hydrolyzes the cholinergic neurotransmitter acetylcholine (ACh) in hepatocytes and displays a simple and widely-used surrogate parameter for hepatic function.[5]

Previous studies have suggested a causative role of BChE in the development of CAD and a predictive value of BChE for cardiac mortality.[3, 6–7] We have recently demonstrated that high BChE activity represents a strong and independent predictive value for long-term outcome in patients with proven CAD and that patients within the lowest tertile of BChE activity had a significantly higher rate of cardiovascular mortality than patients within the remaining tertiles.[4] However, there are yet no data on the age-dependent prognostic value of BChE in younger patients. Lifestyle changes in the western world within the younger population with a higher rate of smokers and obesity in some countries resulted in increased cardiovascular morbidity, such as myocardial infarction in this age group.[8–9] However, these young patients are most often underrepresented in ACS cohort studies. This age group shows a lower mortality rate, but in contrast to older patients the loss of potential life years is much higher. Therefore we aimed to assess the age-dependent prognostic impact of BChE in patients with ACS, which has been previously shown to be inversely associated with mortality.

## Methods

### Study Population

We enrolled 624 ACS patients admitted between December 1996 and December 2009 to the Vienna General Hospital, a tertiary care center with a high-volume cardiac catheterization unit affiliated to the Medical University of Vienna as previously described in detail.([10]) In summary, 208 patients 85 to 100 years (the old age cohort) with a ST elevation MI (STEMI) or a Non-ST elevation MI (NSTEMI) were included in the final analysis and compared to 208 patients 45–64 years (the young cohort) and 208 patients 65–84 years (the middle-aged cohort).[11–12] Groups were matched for gender and time of admission (five-year intervals). A sample size of greater 200 patients per group allowed detection of a risk factor with a risk ratio of 1.4 for cardiac mortality (α = 0.05, power = 80%). The study protocol complies with the declaration of Helsinki and was approved by the local ethics committee of the Medical University of Vienna (EK 159/2011). Informed consent was not given by participants due to the retrospective stetting of this study. Patient records were aonymized and de-identified prior to analysis.

### Data Acquisition and Follow-Up

Patient data were collected from the patients’ electronic medical records by trained chart reviewers and inserted into a predefined data record abstraction form. Standardized variable definitions were used for data acquisition and unclear data were resolved in consensus. Cardiovascular risk factors were defined according to current guidelines.[13] Blood samples were taken at time of admission with ACS before coronary angiography and processed according to local laboratory standards (Department of Laboratory Medicine, Medical University of Vienna, Vienna, Austria). Routine laboratory sampling included measurement of serum BChE activity by enzymatic kinetic assays using butyrylcholine iodide as substrate (Cobas C System CHE2—Roche Diagnostics, Switzerland).

Cardiac mortality was chosen as primary study endpoint as the alternative endpoint all-cause mortality might be influenced by a higher rate of non-cardiac mortality in elderly patients compared to younger patients. The cause of death was assessed by screening the national registry of death until January 2012 (Statistics Austria). Causes of death were defined according to the International Statistical Classification of Disease and Related Health Problems 10^th^ Revision.[14] We chose the ICD-10 codes as follows for our definition of cardiac death: I08, I10, I11, I13, I20, I21, I22, I23, I24, I25, I34, I42, I48 and I50. The autopsy rate within the total cohort was 37.8%.

### Statistical Analysis

Continuous data are shown as median and interquartile range (IQR) in case of non-parametric distribution, categorical parameters as numbers (percentage). Comparisons of continuous data between subgroups were performed using Mann-Whitey-U test or Kruskal-Wallis test. Chi-square test was used to assess the association of categorical data. Cox-Regression hazard analysis was used to assess the influence of BChE activity on cardiac mortality—results were presented as hazard ratio (HR) and the respective 95% confidence interval (CI) per one standard deviation (1-SD) increase for continuous variables. Hazard ratios for BChE were adjusted for potential cofounders due to their association with BChE levels and/or cardiac mortality: age, gender, gamma-GT, quick test, body mass index (BMI), cardio pulmonary resuscitation (CPR) before coronary angiography, hypertension, diabetes mellitus (DM), hypercholesterolemia, positive smoking status, positive family history for CVD, MCI type (STEMI), acute coronary intervention and acute fibrinolysis. The discriminatory power of BChE was confirmed using Harrell’s C-Statistic. Statistical significance was defined by two-sided p-values <0.05. Statistical analyses were performed using the STATA 11 software package (StataCorp LP, USA) and PASW 18.0 (IBM SPSS, USA).

## Results

The baseline characteristics of the 624 ACS patients stratified by age group are shown in Table 1. In the entire cohort, the median age was 73.1 years and 43.1% of patients were male (n = 269). The median BChE concentration was 6.5kU/L (5.6–7.9).

**Table 1:** Baseline characteristics of the entire study-cohort and age strata.

	Total study-cohort	Young patients (45–64a)	Middle-aged patients (65–84a)	Old patients (>85a)	p =
Butyrylcholinesterase, kU/l (IQR)	6.5 (5.6–7.9)	7.2 (5.9–8.6)	6.8 (5.7–8.0)	6.0 (5.0–6.9)	**<0.001**
Age, years (IQR)	73.1 (59.5–85.9)	54.3 (47.4–59.5)	73.1 (68.3–77.6)	87.3 (85.8–89.6)	**<0.001**
Body mass index, kg/m^2^ (IQR)	25.9 (23.8–28.7)	28.1 (24.9–31.7)	26.6 (24.3–28.6)	24.6 (22.7–26.5)	**<0.001**
Gender (male), n (%)	258 (41.3%)	86 (41.3%)	86 (41.3%)	86 (41.3%)	1.000
LDH (max), U/l (IQR)	445.5 (294.2–686.0)	455.0 (294.0–753.0)	471.5 (301.5–706.8)	407.0 (288.3–641.3)	0.359
CK (max), U/l (IQR)	685.0 (249.0–1583.3)	950.0 (320.8–1752.5)	663.0 (252.0–1557.5)	496.0 (191.8–1309.0)	**0.001**
Troponin T (max),μg/l (IQR)	2.2 (0.8–4.9)	2.2 (0.7–4.9)	2.5 (0.9–5.1)	1.9 (0.7–4.7)	0.615
Gamma-GT μkat/l (IQR)	29.0 (19.0–49.0)	30.5 (20.0–53.8)	28.5 (19.0–46.7)	28.0 (17.0–49.0)	0.641
Quick Test % (IQR)	91.5 (79.0–104.8)	96.0 (82.3–109.8)	89.1 (76.3–100.0)	88.0 (73.0–101.8)	**<0.001**
Fibrinogen mg/dl (IQR)	408.5 (347.0–484.5)	388.5 (325.5–457.5)	416.0 (349.3–484.3)	428.0 (358.3–498.8)	**0.001**
AST, U/l (IQR)	47.0 (28.0–106.0)	38.0 (24.0–98.0)	49.0 (28.0–94.0)	49.0 (30.0–116.0)	**0.035**
ALT, U/l (IQR)	27.0 (18.0–45.0)	27.0 (19.0–46.0)	29.0 (19.0–45.0)	25.0 (16.0–39.0)	0.191
Total Bilirubin, μmol/l (IQR)	0.58 (0.41–0.85)	0.49 (0.35–0.70)	0.62 (0.44–0.89)	0.62 (0.46–0.93)	**<0.001**
Resuscitation before angio, n (%)	68 (10.9%)	20 (9.6%)	33 (15.9%)	15 (7.2%)	**0.014**
Hypertension, n (%)	468 (75.0%)	149 (71.6%)	158 (76.0%)	161 (77.4%)	0.368
Diabetes mellitus, n (%)	156 (25.0%)	46 (22.1%)	57 (27.4%)	53 (25.5%)	0.452
Hypercholesterolemia, n (%)	393 (63.0%)	137 (65.9%)	133 (63.9%)	123 (59.1%)	0.342
Current smoker, n (%)	276 (44.2%)	152 (73.1%)	84 (40.4%)	40 (19.2%)	**<0.001**
Family history of CVD, n (%)	222 (35.6%)	90 (43.3%)	62 (29.8%)	70 (33.7%)	**0.013**
STEMI, n (%)	494 (79.2%)	155 (74.5%)	154 (74.0%)	185 (88.9%)	**<0.001**
Coronary intervention, n (%)	455 (72,9%)	178 (85.6%)	158 (76.0%)	119 (57.2%)	**<0.001**
Fibrinolysis, n (%)	67 (10.7%)	34 (16.3%)	23 (11.1%)	10 (4.8%)	**0.001**

Categorial data are presented as counts and percentages, continuous as median and IQR (interquartile range). Categorical data are analyzed using a test for linear association (Maentel–Haenszel-chi-square-test), continuous data using Kruskal-Wallis test.

BChE activity significantly differed between age groups with 7.20 kU/l (IQR 5.90–8.63) in young patients, 6.71 kU/l (IQR 5.70–8.00) in middle-aged patients, and 5.97 kU/l (IQR 5.04–6.90) in old patients (p<0.001). In addition, BChE significantly correlated with gamma-GT (p<0.001, r = 0.23), quick test (p = 0.014, r = 0.17), fibrinogen (p = 0.003, r = 0.20) and body mass index (BMI) (p<0.001, r = 0.25) within young patients. In middle-aged and old patients, BChE activity demonstrated a significant association with maximum creatine kinase values within the hospital-stay (p<0.001, r = 0.20), quick test (p<0.001, r = 0.19), BMI (p<0.001, r = 0.18), as well an inverse association with age (p<0.001, r = -0.29).

Additionally, we detected a significant association of BChE values in young patients with hypertension (p<0.001), diabetes mellitus (p<0.001), hypercholesterolemia (p<0.001), positive smoking history (p<0.001), family history in CVD (p<0.001), STEMI (p<0.001), coronary intervention (p<0.001) and gender (p<0.001) but not in the other age strata. (Table 2). No association of BChE activity was found with home medication of patients or medication during the acute phase of ACS (S1 and S2 Tables)

**Table 2:** Correlation of butyrylcholinesterase activity within young patients and remaining age strata.

	Young patients (45–64 years)			Middle-aged and old patients (65–100 years)			
	values	p =	r =	values	p =	r =	p = *
Butyrylcholinesterase, kU/l (IQR)	7.2 (5.9–8.6)			6.2 (5.4–7.5)			**<0.001**
LHD (max), U/l (IQR)	455.0 (294.0–753.0)	0.968	-0.003	444.5 (296.0–655.0)	0.216	0.061	0.367
CK (max), U/l (IQR)	950.0 (320.8–1752.5)	0.278	0.076	587.5 (225.3–1515.0)	**<0.001**	0.207	0.003
Troponin T (max), μg/l (IQR)	2.2 (0.7–4.9)	0.529	-0.044	2.2 (0.8–4.9)	0.161	0.069	0.957
Gamma-GT μkat/l (IQR)	30.5 (20.0–53.8)	**0.001**	0.238	28.0 (18.0–48.0)	0.108	0.069	0.437
Quick Test % (IQR)	96.0 (82.3–109.8)	**0.014**	0.171	89.0 (75.0–101.0)	**<0.001**	0.196	**<0.001**
Fibrinogen mg/dl (IQR)	388.5 (325.5–457.5)	**0.003**	0.204	421.0 (356.0–496.0)	0.280	-0.053	**<0.001**
Age, years (IQR)	54.3 (47.4–59.5)	0.055	0.134	84.4 (73.1–87.3)	**<0.001**	-0.290	**<0.001**
Body mass index, kg/m^2^ (IQR)	28.1 (24.9–31.7)	**<0.001**	0.252	25.3 (23.4–27.7)	**<0.001**	0.185	**<0.001**
AST, U/l (IQR)	38.0 (24.0–98.0)	0.216	-0.086	49.0 (29.0–106.0)	0.393	-0.042	**0.035**
ALT, U/l (IQR)	27.0 (19.0–46.0)	0.113	0.110	27.0 (17.0–44.0)	**0.037**	-0.103	0.191
Total Bilirubin, μmol/l (IQR)	0.49 (0.35–0.70)	0.057	-0.134	0.62 (0.46–0.91)	**0.001**	-0.168	**<0.001**
**Median BChE values (IQR) within**	**Yes**	**No**		**Yes**	**No**		p = **
Resuscitation before angiography	6.7 (5.3–7.5)	7.3 (5.9–8.7)		6.3 (5.1–8.0)	6.2 (5.5–7.4)		0.747
Hypertension	7.5 (5.9–9.1)	6.8 (5.3–8.0)		6.2 (5.4–7.5)	6.3 (5.5–7.4)		**<0.001**
Diabetes mellitus	8.2 (6.4–9.5)	7.1 (5.9–8.4)		6.4 (5.2–7.3)	6.2 (5.5–7.7)		**<0.001**
Hypercholesterolemia	7.3 (5.9–9.1)	6.8 (5.9–8.4)		6.4 (5.5–7.8)	6.1 (5.1–7.1)		**<0.001**
Current smoker	7.3 (5.9–8.7)	6.7 (5.9–8.5)		6.3 (5.6–7.4)	6.2 (5.4–7.5)		**<0.001**
Family history of CVD	7.2 (6.1–8.7)	7.2 (5.8–8.5)		6.1 (5.3–7.3)	6.3 (5.5–7.5)		**<0.001**
STEMI	7.4 (6.1–8.7)	6.7 (5.4–8.4)		6.2 (5.4–7.5)	6.6 (5.8–7.8)		**<0.001**
Coronary intervention	7.3 (5.9–8.7)	6.9 (5.0–8.4)		6.5 (5.6–7.9)	6.1 (5.2–6.9)		**<0.001**
Fibrinolysis	6.5 (4.8–8.6)	7.3 (6.1–8.6)		5.8 (5.2–7.6)	6.3 (5.5–7.5)		0.759
Gender (male)	7.2 (5.9–8.5)	7.3 (5.9–8.6)		6.3 (5.5–7.7)	6.2 (5.3–7.4)		**0.001**

Continuous data are presented as median (interquartile range) and their association with butyrylcholinesterase was assessed using Spearman–Rho correlation coefficient. Median butyrylcholinesterase values are demonstrated within categorical data and were analyzed using Mann-Whitney-U test

* P-value for comparison of median values within young patients and the remaining age strata using Mann-Whitney-U test

** P-values for comparison of median butyrylcholinesterase values within categorical variables in young patients and the remaining age strata using Mann-Whitney-U test

### Survival analysis

**Fig 1:**
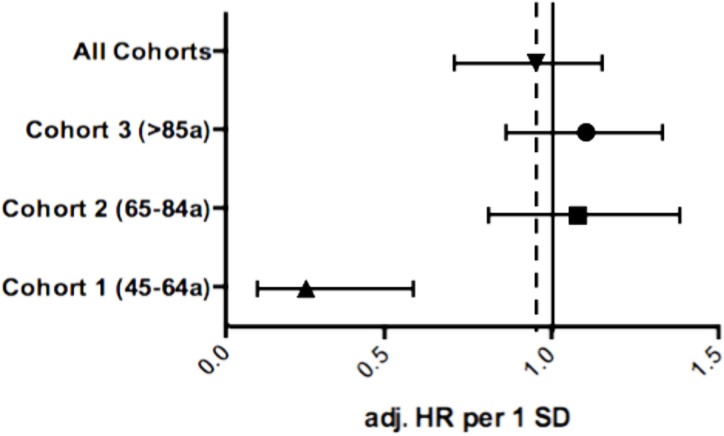
Forrest plot for cardiac mortality stratified by age cohorts: Adjusted HR per 1-SD for butyrylcholinesterase within the age-cohorts (Cohort 1: p = 0.002; Cohort 2: p = 0.612; Cohort 3: p = 0.366) and the entire patient collective (p = 0.608).

After a median follow-up time of 4.0 (IQR 2.0–6.4) years, corresponding to a total of 2682 patient years, 154 (24%) patients died due to a cardiac cause including 11 patients (5%) between 45–64 years, 44 patients (21%) between 65–84 years and 99 patients (47%) between 85–100 years. BChE activity had a strong and independent effect on survival free of cardiac mortality in the entire study cohort with an adjusted HR per one standard deviation (1-SD) of 0.70 (95% CI 0.53–0.93, p = 0.01). In addition, we observed a significant interaction between BChE and age groups (p = 0.008). Subsequent stratification into age groups showed a weaker association of BChE with cardiac mortality with increasing age (Fig 1). In particular, the strongest association of BChE and cardiac mortality after ACS was observed in young patients (45–64 years) with an HR per 1-SD of 0.53 (95% CI 0.33–0.83, p = 0.006). In contrast, BChE showed no association with cardiac mortality both in middle-aged AMI patients (65–84 years) with an HR per 1-SD of 0.65 (95% CI: 0.65–1.10, p = 0.212) and in old patients (85–100 years, HR per 1-SD of 0.99 [95% CI: 0.81–1.21, p = 0.934]). Moreover, interaction term analysis showed a significant modification of the effect of butyrylcholinesterase by age groups (p = 0.008). (Table 3)

**Table 3:** Unadjusted and adjusted effects of BChE on cardiac mortality.

	Total study-cohort		Young patients (45–64a)		Middle-aged patients (65–84a)		Old patients (85–100a)	
	Crude HR	p-value	Crude HR	p-value	Crude HR	p-value	Crude HR	p-value
Unadjusted Effects	0.74 (0.64–0.85)	**<0.001**	0.53 (0.33–0.83)	**0.006**	0.65 (0.65–1.10)	0.212	0.99 (0.81–1.21)	0.934
Adjusted Effects*	0.95 (0.70–1.13)	0.608	0.24 (0.12–0.61)	**0.002**	1.08 (0.81–1.43)	0.612	1.11 (0.89–1.38)	0.366

Cox proportional hazard model for butyrylcholinesterase in the entire cohort and age-groups. Hazard ratios (HR) for continuous variables refer to a 1-SD increase.

* The multivariate model was adjusted for gamma-GT, Quick Test, fibrinogen, age, BMI, resuscitation before angiography, hypertension, diabetes mellitus, hypercholesterinemia, positive smoking status, family history of CVD, STEMI, coronary intervention, fibrinolysis and male gender)

After adjustment for potential cofounders, BChE activity remained a strong and independent prognosticator in young ACS patients (45–64 years) with an adjusted HR per 1-SD of 0.24 (95% CI 0.12–0.61). Similarly, no significant association between BChE and cardiac mortality was found in the multivariate model in middle-aged and old AMI patients (65–84 years: adjusted HR per 1-SD 1.08 [95% CI: 0.81–1.43], p = 0.612; 85–100 years: adjusted HR per 1-SD 1.11[95% CI: 0.89–1.38], p = 0.366). (Table 3)

The C-Statistic for BChE and cardiac mortality was 0.62 for the entire cohort. We were able to demonstrate a decreasing value of C-Statistic by increasing age with 0.75 in young patients, 0.60 in middle-aged patients and 0.51 in old patients.

## Discussion

Our study demonstrates that patients with high BChE activity values have considerably lower cardiac mortality than patients with low BChE activity. Low levels of BChE were a strong and independent risk factor for cardiac mortality in young but not in middle-aged aged and old patients with ACS. Goliasch et al. already demonstrated in a prospective trial in 720 patients with CAD a strong inverse association between BChE activity values with both overall mortality and cardiovascular mortality. They showed that patients in the highest tertile of BChE activity had a significantly lower morality compared with patients in the middle and lower tertile of BChE during a median follow-up of 11.3 years.[4] In addition, Calderon-Margalit et al. demonstrated in a large community-based trial comprising 813 men and 994 women and a long-term follow-up of 10 years, that low BChE activity may be a risk factor for mortality in elderly patients.[3] Furthermore, Distelmaier et al. showed that serum BChE activity is a strong and independent inverse predictor of all-cause and cardiovascular mortality in intensive-care patients undergoing veno-arterial ECMO (Extracorporeal membrane oxygenation) therapy following cardiovascular surgery.[15] In contrast to the above-mentioned studies, which presented and analyzed their data in a non-age related fashion, we stratified our total cohort in age groups. Our data are in accordance with previous results, but expand the current knowledge by this stratification in young, middle-aged and old patients with ACS. While we found a strong association of BChE and cardiac mortality in younger patients (45–64a), the association was weaker in the middle-aged age group (65–84a), and—most interestingly—not present in the old age group (>85a). There were significant differences in patients’ characteristic between our study cohort and those in the trials mentioned before. The mean age of our total cohort was 71.1 years (59.5–85.9), in contrast to a mean age between 62 and 64 in previously mentioned studies.[3–4]

BChE is determined during routine laboratory examination as an indicator for liver dysfunction, since progressive liver damage is associated with compromised serum BChE activity. Interestingly, BChE activity was progressively decreased in elderly patients as compared to the middle-aged and young age group, indicating a moderate decrease in BChE activity with age. Liver damage can be both the cause and the consequence of cardiovascular disease. While the most extreme form of liver damage after ACS—ischemic hepatitis—represent a well-established and clinically relevant consequence of cardiac dysfunction, a causative role of liver damage for cardiovascular morbidity has been described more recently.[16] Especially metabolic liver diseases, such as non-alcoholic steatohepatitis (NASH) can be a driver for cardiac mortality, and represent a new epidemic of liver diseases associated with the increasing prevalence of the metabolic syndrome. Thus, the association of low BChE with higher CV mortality may result from a pre-existing liver disease—as metabolic liver damage and/or NASH share similar risk factors as ACS—that may modulate the course ACS and ultimately cardiac mortality. On the other hand, the lower BChE levels in patients with higher cardiac mortality after ACS might be a surrogate marker for more extensive liver damage in patients with more severe ACS. However, a universal pathophysiological explanation for the observed association with serum BChE activity and cardiac mortality cannot be provided with our clinical association data.

Further associations with laboratory values may support the role of BChE activity as marker of liver function: Calderon-Margalit et al. showed a significant correlation of BChE activity and albumin concentration. This might be caused by reduced binding (and thus reduced stability) of BChE to albumin, or just a parallel surrogate of impaired liver function.[3–4] Unfortunately, albumin levels were not available in our study cohort. However, using quick test (Quick%) as another sensitive marker for liver-function—we found a significant association between BChE activity and Quick%. Additionally, we demonstrated a significant association of BChE and Quick% within the remaining age groups and furthermore a significant difference of Quick% between young ACS and the remaining age strata (p<0.001). This underlines that BChE values may depend on the hepatic synthetic capacity. In contrast, a significant—but weak—correlation between gamma-GT and butyrylcholinesterase in the overall cohort (p = 0.001, r = 0.137) indicates that patients with high gamma-GT values, also have high protective BChE values. High gamma-GT values are a sensitive indicator for hepatic necro-inflammation after metabolic, toxic (alcohol), or cholestatic liver cell damage.[17] However, there was no association of BChE with gamma-GT in middle-aged and old aged subgroups.

In regard to cardiovascular risk factors, younger patients with arterial hypertension show higher values of BChE activity, than both young patients without hypertension and patients within the remaining age strata. In accordance, Goliasch et al. and as well as Alcantara et al. were able to demonstrate a significant association of BChE activity and arterial hypertension.[4,6] Furthermore, several studies demonstrated an association of BChE activity and metabolic risk factors.[3–4] In our study, we could confirm the correlation between BChE and metabolic risk factors, such as body mass index and hypercholesterolemia indicating an association of BChE with lipid metabolism—as reported in previous cohort studies.[3–4] This counterintuitive association of high BChE activity with established cardiovascular risk factors rather suggests a distinct pathophysiological link between low BChE activity and cardiac mortality. While the positive association between BChE activity and cardiovascular risk factors is particularly pronounced in young ACS patients it is unlikely to be the cause of the specific association between low BChE activity and cardiac mortality. The underlying explanation for this specific association with young age needs to be elucidated in future (experimental) studies. However measurement of BChE activity is easily available in routine blood sampling analysis in many countries. It therefore represents an inexpensive and easily available method for risk-prediction in young patients after ACS. In regard of demographic changes and an increasing incidence of younger patients suffering ACS, adequate long-term risk prediction in young patients seems crucial. The measurement of BChE may improve secondary prevention by a closer monitoring and more aggressive secondary prevention in young patients at high risk.

### Limitations

The major limitation represents the retrospective nature of your study. Therefore data about the time delay between index incidence and blood sampling analysis is not available, that might have affected BChE activity values. However, a prospective data acquisition might require a prolonged observation period, due to the low incidence of ACS in young ACS patients. In order to generate sufficient quality of data we tried to collect valid data due to meticulous data processing as described in the method section. Moreover, our study may have a potential selection bias, due to the fact that data have only been collected in a single tertiary care medical center. The high number of STEMI patients within the patient collective may indicate a bias due to selective referral of patients to a tertiary care center. However our patient cohort represents a real-life cohort of ACS patients derived from the non-selected overall population admitted to a tertiary care center due to logistic reasons (available health facility resources) and not based on disease severity.

### Conclusion

Our study confirmed the recently reported association of low serum BChE activity and higher cardiac mortality after ACS. Most importantly, we extended the current knowledge by demonstrating that the prognostic value of BChE levels on mortality in patients with ACS is clearly age-dependent. While BChE activity represents a specific predictor for cardiac mortality in young patients with ACS with an age between 45–64 years, while there is no significant association between cardiac mortality and BChE in other age cohorts. Future studies about BChE and cardiac mortality are required for a better pathophysiological understanding of the age-dependent effect of BChE and for its potential clinical use for tailoring secondary prevention measures.

## Supporting Information

S1 Table Baseline characteristics of the entire study-cohort and age strata: Categorial data are presented as counts and percentages and were analyzed using a test for linear association (Maentel–Haenszel-chi-square-test). (DOCX)

S2 Table Correlation of butyrylcholinesterase activity within young patients and remaining age strata: Median butyrylcholinesterase values are demonstrated within categorical data and were analyzed using Mann-Whitney-U test. * P-values for comparison of median butyrylcholinesterase values within categorical variables in young patients and the remaining age strata using Mann-Whitney-U test
(DOCX)
