# Supplementary material for: Butyrylcholinesterase Predicts Cardiac Mortality in Young Patients with Acute Coronary Syndrome
Source: PLoS One. 2015 May 1;10(5):e0123948. doi: 10.1371/journal.pone.0123948 (PMC4416767; doi:10.1371/journal.pone.0123948)
Supplement: S1 Table — (DOCX) [file pone.0123948.s001.docx]

**Supplemental table 1: Baseline characteristics of the entire study-cohort and age strata**

|  | **Total study-cohort** | **Young patients (45-64a)** | **Middle-aged patients (65-84a)** | **Old patients (>85a)** | **p=** |
| --- | --- | --- | --- | --- | --- |
| ASA before event | 190 (30.4) | 31 (14.9) | 57 (27.4) | 102 (49.0) | **<0.001** |
| Beta-blockers before event | 164 (26.3) | 45 (21.6) | 53 (25.5) | 66 (31.7) | 0.062 |
| Statins before event | 122 (16.6) | 24 (11.5) | 53 (25.5) | 45 (21.6) | **0.001** |
| ACE/ATII-inhibitor before event | 421 (67.5) | 157 (75.5) | 142 (68.3) | 122 (58.7) | **0.001** |
| ASA loading | 462 (74.0) | 193 (92.8) | 158 (76.0) | 111 (53.4) | **<0.001** |
| Clopidogrel loading | 380 (61.4) | 160 (77.2) | 132 (64.7) | 88 (42.3) | **<0.001** |
| Heparin loading | 507 (81.3) | 192 (92.3) | 173 (83.2) | 142 (68.3) | **<0.001** |

**Supplemental table 1: Categorial data are presented as counts and percentages and were analyzed using a test for linear association (Maentel–Haenszel-chi-square-test).**
