# Supplementary material for: Butyrylcholinesterase Predicts Cardiac Mortality in Young Patients with Acute Coronary Syndrome
Source: PLoS One. 2015 May 1;10(5):e0123948. doi: 10.1371/journal.pone.0123948 (PMC4416767; doi:10.1371/journal.pone.0123948)
Supplement: S2 Table — * P-values for comparison of median butyrylcholinesterase values within categorical variables in young patients and the remaining age strata using Mann-Whitney-U test (DOCX) [file pone.0123948.s002.docx]

**Supplemental table 2: Correlation of butyrylcholinesterase activity within young patients and remaining age strata**

|  | **Young patients (45-64 years)** | | | **Middle-aged and old patients (65-100 years)** | | | |
| --- | --- | --- | --- | --- | --- | --- | --- |
| **Median BChE values (IQR) within** | **Yes** | **No** | **p=** | **Yes** | **No** | **p=** | **p=*** |
| Butyrylcholinesterase, kU/l (IQR) | 7.2 (5.9-8.6) |  |  | 6.2 (5.4-7.5) |  |  | **<0.001** |
| ASA before event | 7.9 (5.8-9.5) | 7.1 (5.9-8.5) | 0.334 | 6.1 (5.3-7.1) | 6.3 (5.5-7.7) | **0.046** | **0.001** |
| Beta-blockers before event | 7.3 (5.9-9.1) | 7.2 (5.9-8.5) | 0.577 | 6.2 (5.3-7.6) | 6.3 (5.5-7.5) | 0.446 | **0.001** |
| Statins before event | 8.0 (5.9-9.5) | 7.2 (5.9-8.5) | 0.192 | 6.3 (5.4-7.4) | 6.2 (5.4-7.5) | 0.433 | **0.001** |
| ACE/ATII-inhib. before event | 7.4 (5.9-8.7) | 6.6 (5.9-8.4) | 0.062 | 6.3 (5.5-7.7) | 6.2 (4.9-7.3) | 0.274 | **<0.001** |
| ASA loading | 7.3 (5.9-8.7) | 6.5 (5.4-8.3) | 0.091 | 6.3 (5.6-7.6) | 6.0 (4.9-7.3) | **0.029** | **<0.001** |
| Clopidogrel loading | 7.4 (6.2-8.7) | 6.5 (4.7-8.2) | **0.005** | 6.4 (5-6-7.6) | 6.0 (5.0-7.3) | **0.013** | **<0.001** |
| Heparin loading | 7.3 (6.1-8.6) | 5.8 (5.0-7.8) | **0.033** | 6.4 (5.5-7.7) | 5.9 (4.8-6.6) | **<0.001** | **<0.001** |

**Supplemental table 2: Median butyrylcholinesterase values are demonstrated within categorical data and were analyzed using Mann-Whitney-U test**

*** P-values for comparison of median butyrylcholinesterase values within categorical variables in young patients and the remaining age strata using Mann-Whitney-U test**
